# Metabolic crosstalk between differentiated thyroid cancer and cardiovascular disease: insulin resistance as a molecular bridge—a proposed mechanism

**DOI:** 10.3389/fendo.2026.1795263

**Published:** 2026-04-22

**Authors:** Guizhang Hou, Tianshu Gao

**Affiliations:** 1 Liaoning University of Traditional Chinese Medicine, Shenyang, China; 2 Affiliated Hospital of Liaoning University of Traditional Chinese Medicine, Shenyang, China

**Keywords:** cardiovascular risk, differentiated thyroid cancer, insulin resistance, metabolic reprogramming, thyroid cancer

## Abstract

Differentiated thyroid cancer (DTC) is generally associated with favorable survival, yet long-term survivors may experience an increased burden of cardiovascular disease (CVD). This risk has often been attributed primarily to TSH suppression therapy, although metabolic factors might also contribute. In this article, we propose a conceptual DTC–IR–CVD axis, in which insulin resistance (IR) may represent one potential molecular link between thyroid malignancy and cardiovascular vulnerability. Available evidence supports associations between IR, diabetes, thyroid tumorigenesis, and CVD; however, direct clinical evidence that DTC itself induces systemic IR remains limited. We therefore discuss a hypothesis whereby tumor-related metabolic reprogramming, inflammatory signaling, extracellular vesicle communication, and treatment-related endocrine perturbations may together contribute to endothelial dysfunction, arterial stiffness, and myocardial remodeling in selected patients. We further outline how this framework could inform future cardio-oncologic risk stratification and generate testable translational hypotheses. Overall, the proposed model should be viewed as a hypothesis-generating framework rather than an established causal pathway.

## 1 Introduction

Differentiated thyroid cancer (DTC) is one of the most rapidly increasing endocrine malignancies worldwide (1). However, a substantial proportion of the observed rise in incidence is widely considered to reflect overdiagnosis, particularly the increased detection of small, low-risk papillary thyroid cancers through intensified imaging and surveillance (2, 3). While overall survival rates are excellent, the long-term management of DTC is increasingly complicated by a significant burden of metabolic and cardiovascular comorbidities (4). Traditionally, cardiovascular risk in DTC has been attributed almost exclusively to the hemodynamic effects of TSH suppression therapy (5). However, current evidence more consistently supports insulin resistance (IR) and diabetes mellitus (DM) as potential risk factors for thyroid carcinogenesis, while the extent to which DTC itself contributes to systemic IR remains less clearly established (6–8). Although IR appears to be a plausible contributor to thyroid tumorigenesis, direct evidence that DTC itself induces systemic IR in patients remains limited. We therefore present the DTC–IR–CVD axis as a hypothesis-generating framework rather than an established bidirectional model (9). In this article, we propose a conceptual framework linking DTC, insulin resistance, and cardiovascular risk. We discuss the molecular and clinical observations that may support such a framework, highlight alternative explanations, and outline key knowledge gaps that must be addressed before causal inferences can be made. Rather than establishing DTC as a systemic metabolic disorder, this model is intended as a hypothesis-generating perspective for future mechanistic and clinical studies (10).

## 2 Intratumoral metabolic reprogramming: the engine of systemic dysfunction

### 2.1 Oncogenic drivers of the glycolytic shift

Unlike general solid tumors, the metabolic plasticity of Differentiated Thyroid Cancer (DTC) is intrinsically linked to its endocrine origins and specific mutational landscape. The transition from a localized thyroid lesion to a metabolically active malignancy is thought to be driven, at least in part, by oncogenic alterations such as BRAF V600E and RAS mutations. These oncogenes constitutively activate the MAPK and PI3K/Akt signaling cascades, stabilizing HIF-1α to orchestrate a fundamental shift towards aerobic glycolysis (the Warburg effect) (11, 12). This reprogramming involves the coordinated upregulation of GLUT1 transporters and hexokinase-2 (HK2), facilitating rapid glucose influx to support proliferation (13). Crucially, this glycolytic surge has a specific “thyroid cost”: the repression of the sodium-iodide symporter (NIS), which underpins the radioiodine refractoriness often seen in metabolically active tumors (14). Furthermore, this high-energy demand creates a “metabolic sink” effect, sequestering systemic nutrients while generating lactate via LDHA upregulation. In thyroid cancer, increased glycolytic flux is associated with enhanced lactate production and tumor aggressiveness; however, the downstream effects of lactate-mediated stromal remodeling have been characterized more extensively in other solid tumors than in DTC specifically (15, 16).

### 2.2 Lipid remodeling and mitochondrial inflexibility

While glucose provides the fuel for rapid growth, lipid reprogramming ensures survival under stress. In cancer more broadly, SREBP-dependent lipogenic programs support membrane biogenesis and anabolic growth. In thyroid cancer, available data suggest that SREBP1 and FASN are upregulated and may be associated with more aggressive biological behavior, although the mechanistic evidence remains more limited than in some other tumor types (17). Concurrently, there is a paradoxical upregulation of fatty acid oxidation (FAO) via CPT1A, providing an alternative ATP source when glucose is scarce. Central to this dysregulation is mitochondrial inflexibility. MAPK signaling promotes Drp1-mediated mitochondrial fission (specifically via Ser616 phosphorylation), leading to a fragmented mitochondrial network that is bioenergetically inefficient (18). This compromise in the electron transport chain results in elevated reactive oxygen species (ROS) production, creating a self-reinforcing “ROS-mtDNA loop” that induces genomic instability and further suppresses thyroid differentiation markers (19). These intracellular alterations generate metabolic byproducts—including oxidized lipids and mitochondrial DNA fragments—that spill over into the systemic circulation, acting as damage-associated molecular patterns (DAMPs) that trigger systemic inflammation (20).

## 3 Potential molecular links between DTC and systemic insulin resistance

### 3.1 Tumor-Derived inflammation and insulin signaling blockade

One hypothesized mechanism by which DTC may influence systemic insulin sensitivity extends beyond nutrient competition and involves tumor-associated inflammatory signaling. Thyroid tumors have been reported to exhibit a pro-inflammatory microenvironment, and tumor-associated cytokine signaling may contribute to systemic metabolic perturbation; however, the extent to which this translates into clinically meaningful whole-body insulin resistance remains uncertain (21, 22). These mediators travel to peripheral metabolic tissues—primarily skeletal muscle and liver—where they trigger the aberrant serine phosphorylation (specifically at Ser307/312) of Insulin Receptor Substrate-1 (IRS-1) (23). This pathological phosphorylation event mechanistically uncouples the insulin receptor from the PI3K-Akt pathway, effectively locking the GLUT4 transporter inside the cell. This inflammatory pattern resembles mechanisms described in type 2 diabetes and obesity-related insulin resistance, in which cytokine signaling can impair IRS–PI3K–Akt signaling; whether comparable tumor-associated processes produce sustained systemic insulin resistance in DTC survivors remains to be established (23, 24).

### 3.2 Exosomal miRNAs: a novel vector of inter-organ crosstalk

Beyond soluble cytokines, a more sophisticated mechanism of communication involves tumor-derived exosomes. These nano-vesicles, shed abundantly by aggressive DTC cells, act as “Trojan horses” carrying oncogenic miRNAs—specifically miR-21, miR-146b, and miR-221/222—to distant organs (25). Upon internalization by adipocytes and myocytes, these miRNAs directly target and suppress key insulin signaling components, including PTEN and PPARγ (26, 27). Recent studies have confirmed that miR-146b can actively remodel adipose tissue towards a pro-inflammatory phenotype, demonstrating that the tumor can genetically reprogram host metabolism to ensure nutrient availability (28).

### 3.3 Endocrine disruptions: adipokines and the IGF axis

**Figure 1:**
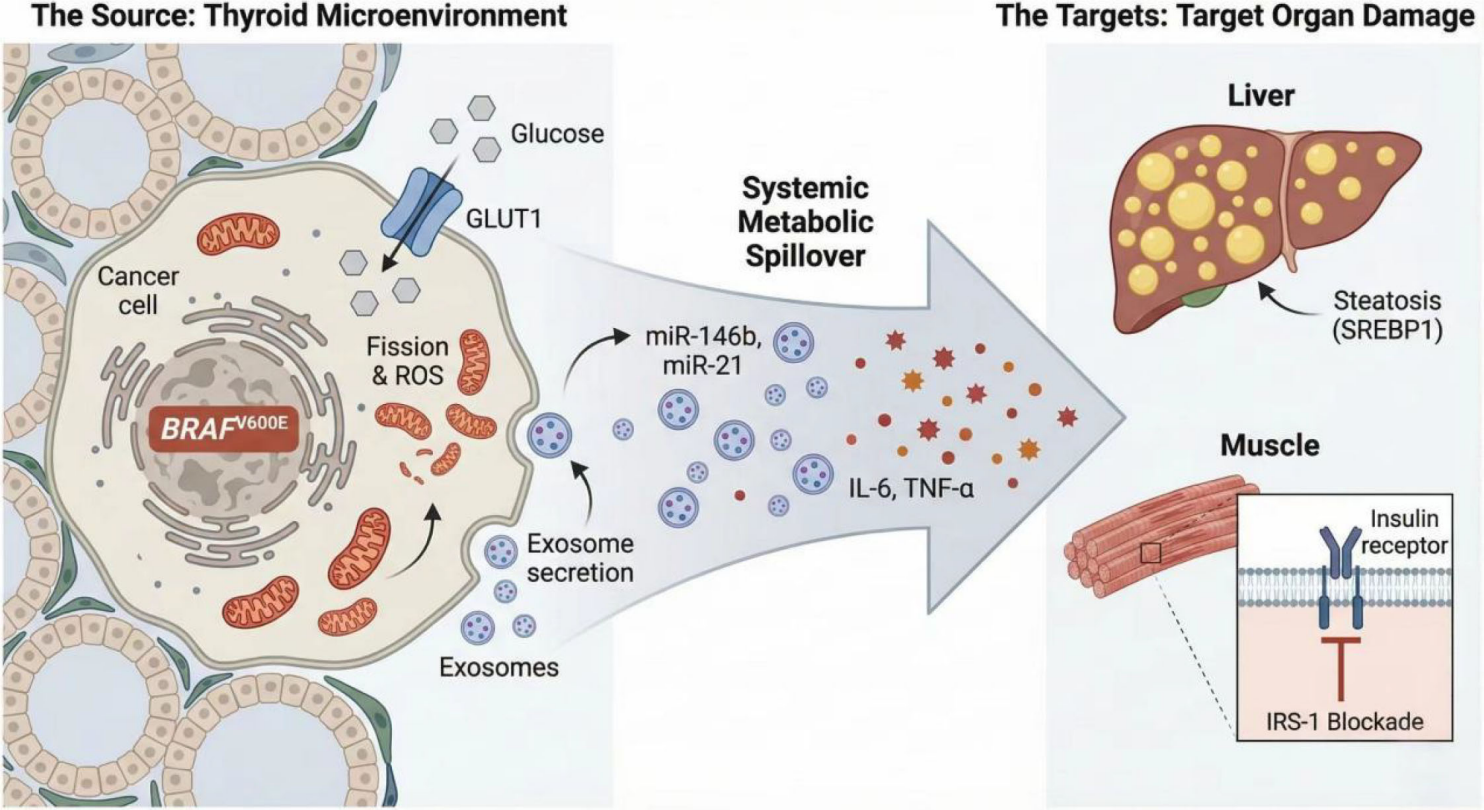
Proposed conceptual links between thyroid tumor metabolism, insulin resistance, and cardiovascular vulnerability. (Left) The thyroid cancer microenvironment driven by the BRAF V600E mutation. Oncogenic signaling upregulates GLUT1 and Hexokinase-2 (HK2), promoting the Warburg effect (aerobic glycolysis) while suppressing NIS expression. Mitochondrial fission and dysfunction lead to ROS accumulation. (Middle) The “Metabolic Spillover.” The tumor may release pro-inflammatory cytokines (IL-6, TNF-α) and oncogenic exosomes carrying miRNAs (e.g., miR-146b, miR-21) into the systemic circulation. (Right) Potential peripheral metabolic effects. In the liver, these signals may contribute to lipogenesis and steatosis via SREBP1 activation. In skeletal muscle, they trigger aberrant phosphorylation of IRS-1, blocking insulin receptor signaling. Created with BioRender.com and finalized by the authors.

The metabolic disarray is further compounded by a skewed endocrine milieu. Studies of thyroid carcinoma have reported associations between altered adipokine profiles—particularly higher leptin, TNF-α, and IL-6—and thyroid cancer biology, whereas evidence for reduced adiponectin is less consistent. These observations may reflect links between obesity-related metabolic dysfunction and thyroid tumorigenesis, rather than demonstrating that DTC itself induces a systemic adipokine imbalance (29, 30). Additionally, the upregulation of the IGF-1/IGF-1R axis in DTC creates a complex feedback loop. While IGF-1 amplifies mitogenic signaling for the tumor, its overexpression desensitizes metabolic pathways through crosstalk with insulin receptors (31–33). These pathways may represent one potential mechanism contributing to downstream cardiovascular vulnerability, but current evidence is insufficient to define them as a central pathological feature across DTC survivors (**Figure 1**).

## 4 The clinical consequences: from molecular mechanisms to risk stratification

### 4.1 Vascular collision: endothelial insulin resistance and atherogenic dyslipidemia

In the vascular endothelium, insulin resistance creates a specific signaling dichotomy. While the protective PI3K/Akt/eNOS pathway is blunted by inflammatory cytokines—leading to reduced nitric oxide bioavailability—the MAPK/ERK pathway remains hyper-responsive to compensatory hyperinsulinemia (34). This selective insulin resistance drives the production of endothelin-1 and adhesion molecules (ICAM-1, VCAM-1), creating a pro-constrictive and pro-inflammatory milieu (35). This endothelial vulnerability may be further aggravated by atherogenic dyslipidemia. In insulin-resistant states, hyperinsulinemia can activate hepatic SREBP-1c and promote VLDL overproduction; similar lipogenic pathways have also been described in cancer metabolism more broadly, including thyroid cancer, although their direct contribution to vascular injury in DTC survivors remains to be defined (36–38). Consequently, the vasculature loses its ability to dilate in response to stress and becomes susceptible to rapid plaque formation and instability, laying the groundwork for acute cardiovascular events distinct from classic metabolic syndrome (39).

### 4.2 A Potential two-hit model: residual metabolic vulnerability and TSH suppression

**Figure 2:**
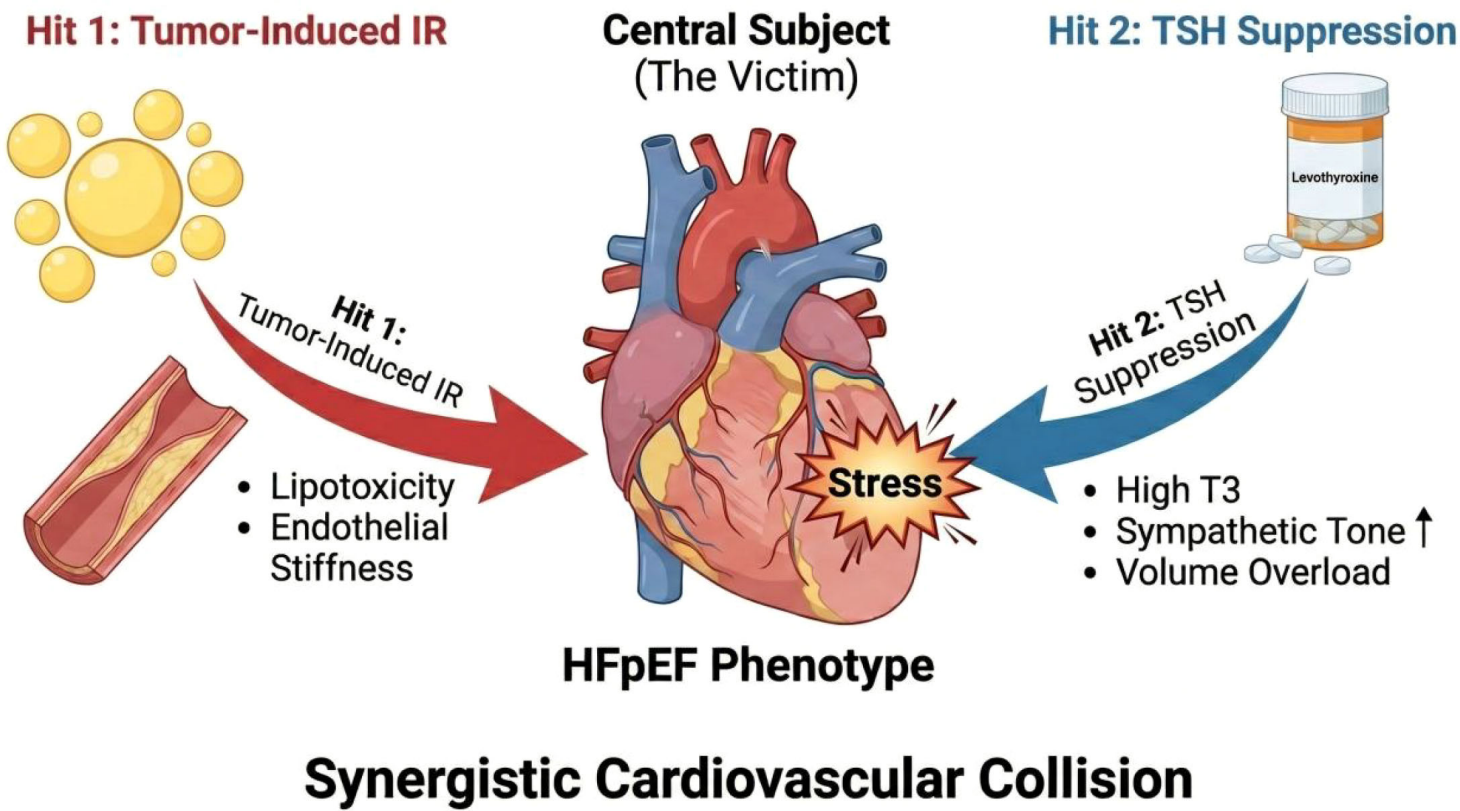
A proposed two-hit model linking baseline cardiometabolic vulnerability and TSH suppression-related cardiovascular stress. A schematic illustration of how baseline cardiometabolic vulnerability and TSH-suppressive therapy may converge to increase cardiovascular stress in selected survivors. Hit 1 (Metabolic Vulnerability): insulin resistance, obesity-related metabolic dysfunction, dyslipidemia, and endothelial impairment. Hit 2 (Hemodynamic Stress): exogenous subclinical hyperthyroidism increases sympathetic tone, heart rate, and myocardial oxygen demand. Outcome: their interaction may promote left ventricular remodeling and diastolic dysfunction in vulnerable survivors. Created with BioRender.com and finalized by the authors.

In the post-operative setting, cardiovascular risk in differentiated thyroid cancer (DTC) survivors may be more plausibly interpreted as the interaction between residual cardiometabolic vulnerability and TSH-suppressive therapy, rather than as a persistent tumor-driven myocardial process. Insulin resistance, obesity-related metabolic dysfunction, and atherogenic dyslipidemia can impair myocardial metabolic flexibility, favoring abnormal fatty-acid handling, lipotoxic stress, oxidative stress, and diastolic dysfunction (40, 41). Against this background, long-term TSH suppression may impose an additional hemodynamic burden by increasing heart rate, myocardial contractility, and oxygen demand (5, 42). We therefore propose a hypothesis-generating two-hit model in which persistent metabolic abnormalities constitute the first hit and suppression-related thyrotoxic stress constitutes the second hit. Importantly, this model does not assume that active tumor tissue persists in most survivors; rather, it may be most relevant in patients with pre-existing insulin resistance, obesity, dyslipidemia, or hypertension. This interpretation should also be viewed in light of contemporary practice, as the 2025 ATA guidelines support more individualized TSH targets and less routine prolonged aggressive suppression in lower-risk patients (43). Thus, residual metabolic vulnerability may modify the cardiovascular cost of TSH suppression in selected DTC survivors, although direct prospective evidence remains limited (44) (**Figure 2**).

### 4.3 A potential cardiometabolic layer within contemporary risk-adapted follow-up

Current ATA-based management already incorporates dynamic response-to-therapy assessment when guiding follow-up intensity and TSH targets after primary treatment for differentiated thyroid cancer (DTC) (43, 45). Within this contemporary framework, we suggest that cardiometabolic profiling may serve as a complementary layer rather than a replacement for guideline-based stratification. Specifically, indices reflecting insulin resistance and metabolic resilience, together with selected vascular or cardiac markers, may help identify survivors who are less likely to tolerate prolonged or intensive TSH suppression (46, 47). Accordingly, the present concept should be viewed not as a new stand-alone paradigm, but as a hypothesis-generating extension of current response-to-therapy–based care. In this context, metabolic vulnerability may help inform the individualized balance between oncologic control and long-term cardiovascular safety. This interpretation is also consistent with the evolving shift toward more individualized TSH targets and less routine prolonged aggressive suppression in lower-risk patients (43, 45) (**Table 1**).

**Table 1:** Proposed cardiometabolic considerations that may complement current ATA-based TSH decision-making.

Patient category	Oncologic risk (ATA)	Metabolic-cardio burden (New Layer)	Potential TSH consideration	Rationale & monitoring
Group A: Higher Oncologic Priority	High/Intermediate	Any (Low or High)	< 0.1 mIU/L *(when clinically indicated)*	Priority: Oncologic control remains the primary consideration. In such settings, metabolic burden may warrant closer cardiovascular and skeletal monitoring rather than major deviation from guideline-based suppression. Monitor: ECG, heart rate, bone density.
Group B: Increased Cardiometabolic Vulnerability	Low/Intermediate	High • HOMA-IR > 2.5 • Visceral Obesity • E/e’ ratio > 14 (Diastolic Dysfunction)	0.1 – 0.5 mIU/L *(may be considered in selected patients)*	Priority: Individualized balance. In survivors with lower oncologic risk but higher cardiometabolic vulnerability, less intensive suppression may be discussed within the current response-to-therapy framework to reduce cumulative cardiovascular burden. Monitor: HOMA-IR, blood pressure, echocardiography, lipids.
Group C: Lower Cardiometabolic Burden	Low	Low • Insulin Sensitive • Normal BMI • Normal Endothelial Function	0.5 – 2.0 mIU/L *(may be appropriate depending on response-to-therapy status)*	Priority: Long-term safety and routine survivorship follow-up. In lower-risk survivors with lower cardiometabolic burden, management may align with contemporary guideline trends toward less aggressive long-term suppression. Monitor: Routine TSH, thyroglobulin, standard follow-up parameters.

ATA, American Thyroid Association; HOMA-IR, Homeostatic Model Assessment for Insulin Resistance; E/e’, Ratio of early transmitral flow velocity to early diastolic mitral annular velocity (marker of diastolic pressure).

This table is intended as a hypothesis-generating framework that may complement, rather than replace, current ATA response-to-therapy–based management. It is designed to illustrate how cardiometabolic features such as insulin resistance, visceral adiposity, and subclinical cardiac vulnerability might inform individualized discussion of TSH suppression intensity in selected survivors. These categories should not be interpreted as formal guideline recommendations.

## 5 Therapeutic and translational perspectives of the DTC–IR–CVD axis

### 5.1 Individualized TSH suppression in the context of cardiometabolic risk

TSH suppression remains an important component of DTC management, particularly in patients with persistent structural disease, biochemical evidence of disease, or higher recurrence risk (43, 45). However, its intensity and duration are now increasingly individualized because long-term subclinical thyrotoxicosis may impose clinically relevant cardiovascular and skeletal harm (43, 48, 49). According to the 2025 ATA guidelines, the potential benefit of stronger suppression is expected mainly in selected higher-risk or progressive disease settings, whereas in lower-risk patients the balance may shift toward harm (43, 45). Recognized adverse effects include worsening angina in patients with ischemic heart disease, atrial fibrillation, stroke, increased cardiovascular mortality, and osteoporosis or fractures, particularly in postmenopausal women (43, 50). Against this background, our proposal is not to replace the current ATA response-to-therapy framework. Rather, we suggest that cardiometabolic profiling may help refine tolerance assessment in selected survivors when determining how aggressively and how long TSH should be suppressed (43, 51, 52). This concept should therefore be regarded as a possible complement to current guideline-based care rather than a separate management algorithm (**Table 1**).

### 5.2 Repurposed metabolic agents as hypothesis-generating adjuncts in selected survivors

Beyond adjustment of thyroid hormone therapy, repurposed metabolic agents may merit investigation as adjunctive strategies in selected DTC survivors with obesity, insulin resistance, diabetes, or heightened cardiovascular risk. Metformin is of interest because it improves insulin sensitivity and may influence AMPK-related growth signaling; in thyroid cancer, its potential antitumor and redifferentiation effects remain suggestive rather than established in routine clinical practice (53–55). GLP-1 receptor agonists may be relevant primarily through weight reduction and broader cardiometabolic risk improvement in survivors with obesity or diabetes, whereas SGLT2 inhibitors may be particularly relevant in those with diabetes, chronic kidney disease, or heart failure risk because of their established cardiovascular, renal, and metabolic benefits (56–60). However, direct evidence supporting the routine use of these agents specifically to modify DTC-related outcomes is lacking (55). Their integration should therefore be viewed as a hypothesis-generating survivorship strategy for selected metabolically vulnerable patients rather than as a cornerstone of a new treatment paradigm.

### 5.3 Lifestyle as medicine: impact of dietary interventions on the DTC–IR–CVD Axis

#### 5.3.1 Adiposity and diet as modifiers of survivorship cardiometabolic risk

In the survivorship setting, adiposity and dietary pattern are relevant primarily because they shape overall cardiometabolic risk rather than because they directly reflect ongoing tumor-driven signaling (43, 61). Visceral adiposity promotes low-grade inflammation, insulin resistance, dyslipidemia, and hepatic steatosis, all of which are established contributors to cardiovascular disease and may also be associated with less favorable outcomes in thyroid cancer populations (29, 30, 46, 61). Accordingly, dietary and weight-management strategies may be best viewed as general risk-reduction measures in selected DTC survivors, especially those with obesity or metabolic dysfunction, rather than as evidence-based interventions targeting a persistent tumor-derived axis after surgery. This distinction is particularly important because not all DTC survivors are insulin resistant, and the clinical relevance of this pathway is likely to be greatest in metabolically vulnerable subgroups.

#### 5.3.2 Metabolic pathways potentially relevant to survivorship care

Dietary interventions may influence insulin/IGF-1 signaling primarily by reducing glycemic load, thereby improving insulin sensitivity and reducing chronic hyperinsulinemia (62). Concurrently, anti-inflammatory diets rich in omega-3 fatty acids may improve adiponectin-related signaling and help preserve endothelial function in insulin-resistant states (63). Additionally, fiber-rich diets can remodel the gut microbiota to increase short-chain fatty acid (SCFA) production, which has been associated with improved insulin sensitivity and broader cardiometabolic homeostasis (64, 65). In the post-operative setting, these pathways are best interpreted as general metabolic mechanisms relevant to selected DTC survivors with obesity, insulin resistance, or cardiovascular vulnerability, rather than as evidence of persistent tumor-driven signaling after tumor removal.

#### 5.3.3 Practical dietary patterns in survivorship

Practical dietary patterns may be relevant to DTC survivorship primarily through their effects on body weight, insulin sensitivity, and overall cardiovascular risk. Among these, Mediterranean-style dietary patterns are the most clinically plausible because they are associated with improved metabolic indices and may be easier to integrate into long-term survivorship care (62, 66). By contrast, more restrictive approaches such as intermittent fasting remain insufficiently studied in DTC survivors and should be considered exploratory rather than evidence-based recommendations (67).

#### 5.3.4 Synergistic effects of physical activity and TSH suppression management

Physical activity acts as a potent insulin-sensitizing intervention by enhancing skeletal muscle glucose uptake and improving overall metabolic health (68). In cancer survivorship more broadly, regular aerobic exercise has been associated with improved cardiometabolic fitness, reduced fatigue, and better quality of life, and similar supportive benefits are plausible in selected DTC survivors (69, 70). Exercise may also be relevant in the context of TSH suppression because improved cardiovascular conditioning and autonomic balance could theoretically reduce tolerance issues related to tachycardia or reduced exercise capacity; however, this specific benefit in DTC survivors remains to be established. When combined with dietary measures, physical activity may therefore be viewed as a general supportive strategy for survivors with obesity, insulin resistance, or elevated cardiovascular risk, rather than as an intervention targeting a persistent tumor-driven axis.

### 5.4 Current limitations and knowledge gaps

**Figure 3:**
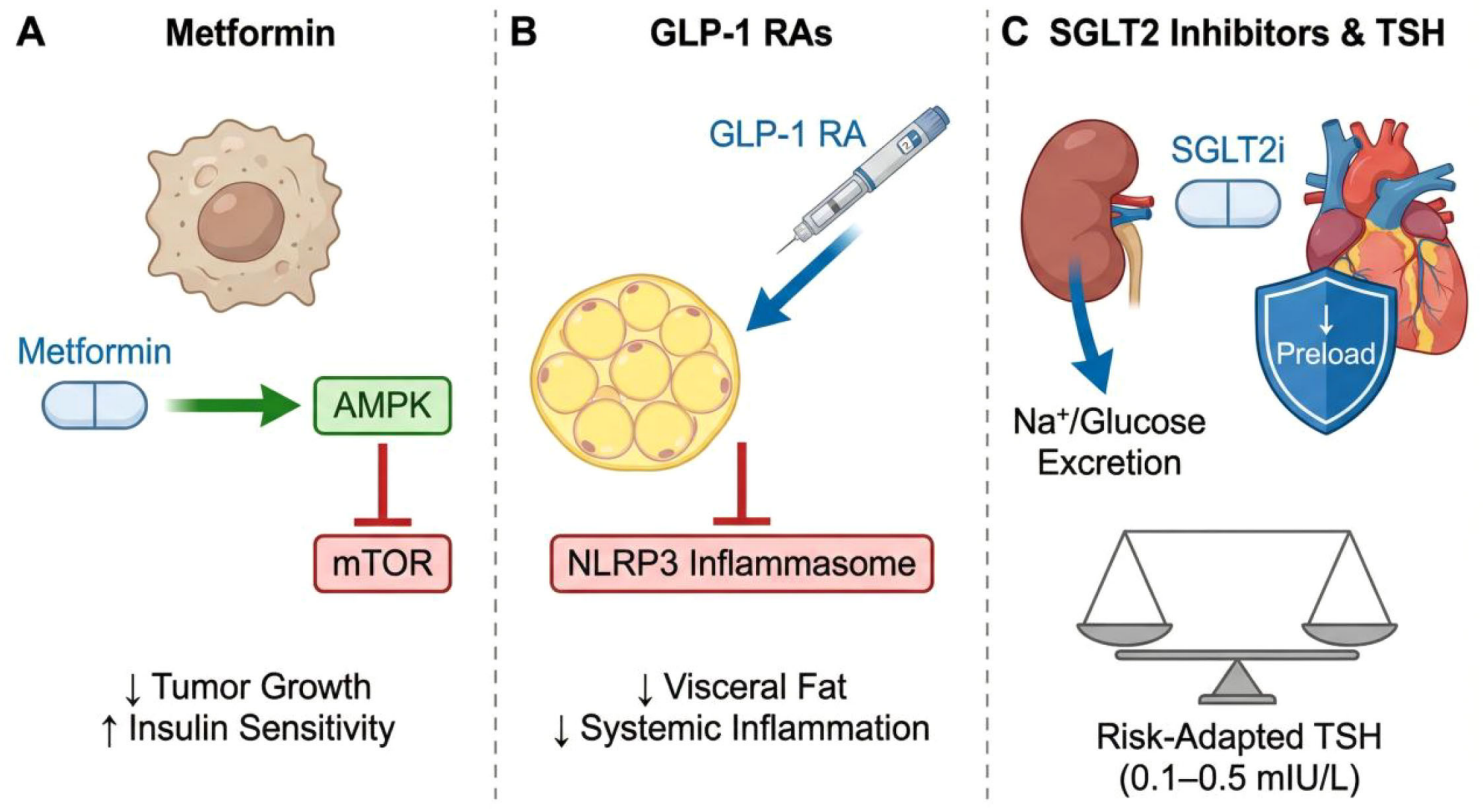
Hypothesis-generating framework for integrating cardiometabolic features into survivorship discussions of TSH suppression intensity. **(A)** Metformin may improve insulin sensitivity and could have adjunctive metabolic relevance in selected survivors; potential antitumor effects in thyroid cancer remain exploratory. **(B)** GLP-1 receptor agonists may reduce visceral adiposity and improve broader cardiometabolic risk profiles in selected survivors. **(C)** SGLT2 inhibitors may provide cardiovascular, renal, and metabolic benefit in selected survivors with diabetes, chronic kidney disease, or heart failure risk. This panel also illustrates a hypothesis-generating, risk-adapted approach to individualized discussion of TSH suppression intensity within current guideline-based survivorship care. Created with BioRender.com and finalized by the authors.

The present manuscript should be interpreted as a hypothesis-generating conceptual framework rather than a demonstration of a proven causal pathway. First, much of the evidence linking thyroid cancer, insulin resistance, and cardiovascular risk is indirect, cross-sectional, or extrapolated from non-thyroid cancer biology and general cardiometabolic literature (71). Second, direct clinical evidence that DTC itself induces persistent systemic insulin resistance after surgery is currently lacking. An alternative and in many settings more plausible explanation is that insulin resistance and related metabolic dysfunction may independently contribute both to thyroid tumorigenesis and to cardiovascular disease, without requiring a tumor-driven causal axis. Third, the proposed interaction between metabolic vulnerability and TSH suppression remains inferential, because prospective studies simultaneously tracking metabolic markers, suppression intensity, and cardiovascular outcomes in DTC survivors are limited (48, 72). Accordingly, the mechanisms discussed throughout this manuscript should be regarded as proposed hypotheses requiring validation rather than established conclusions (**Figure 3**).

## 6 Conclusion

### 6.1 A conceptual framework rather than a definitive disease model

The literature reviewed here supports the clinical importance of considering metabolic and cardiovascular comorbidity in patients with differentiated thyroid cancer (DTC), but it does not establish DTC as a proven systemic metabolic disorder. Rather, the proposed DTC–IR–CVD axis should be viewed as a conceptual framework intended to organize a set of potentially interacting mechanisms. Importantly, an alternative explanation must also be considered: insulin resistance and related metabolic dysfunction may independently contribute both to thyroid tumorigenesis and to cardiovascular disease, without requiring a direct tumor-driven systemic axis. In this sense, the present model is best understood as a hypothesis to be tested, not as a settled biological conclusion.

### 6.2 Clinical relevance in the post-operative setting

In the post-operative setting, the clinical relevance of the proposed model likely lies less in persistent tumor-derived signaling and more in the interaction between residual cardiometabolic vulnerability and long-term endocrine management. Some survivors may enter follow-up with obesity, insulin resistance, dyslipidemia, hypertension, or reduced cardiovascular reserve, and these factors may influence tolerance to TSH suppression (48, 72). Accordingly, the previously discussed “two-hit” idea is more cautiously interpreted here as the coexistence of baseline metabolic vulnerability and treatment-related thyrotoxic stress, rather than as evidence that surgically removed tumors continue to drive systemic injury in most survivors.

### 6.3 Future research directions

Future work should determine whether metabolic profiling can meaningfully improve long-term risk stratification in DTC survivorship beyond existing oncologic frameworks. Prospective studies are needed to clarify which survivors, if any, exhibit persistent insulin resistance or cardiovascular vulnerability that is clinically relevant to TSH suppression decisions. Likewise, the potential role of repurposed metabolic therapies and structured lifestyle interventions should be examined in carefully phenotyped cohorts rather than inferred from broader cardiometabolic literature alone. At present, these ideas are best considered research hypotheses that may help shape a more integrated survivorship agenda.

## References

[1] Sung H Ferlay J Siegel RL Laversanne M Soerjomataram I Jemal A . Global cancer statistics 2020: GLOBOCAN estimates of incidence and mortality worldwide for 36 cancers in 185 countries. CA Cancer J Clin. (2021) 71:209–49. doi: 10.3322/caac.21660. PMID: 33538338

[2] Chen DW Haymart MR . Unravelling the rise in thyroid cancer incidence and addressing overdiagnosis. Nat Rev Endocrinol. (2026) 22:10–20. doi: 10.1038/s41574-025-01168-y. PMID: 40903496 PMC12814954

[3] Welch HG Doherty GM . Saving thyroids - overtreatment of small papillary cancers. N Engl J Med. (2018) 379:310–2. doi: 10.1056/nejmp1804426. PMID: 30044933

[4] Suh B Shin DW Park Y Lim H Yun JM Song SO . Increased cardiovascular risk in thyroid cancer patients taking levothyroxine: a nationwide cohort study in Korea. Eur J Endocrinol. (2019) 180:11–20. doi: 10.1530/eje-18-0551. PMID: 30400044

[5] Biondi B Cooper DS . Thyroid hormone suppression therapy. Endocrinol Metab Clin North Am. (2019) 48:227–37. doi: 10.1016/j.ecl.2018.10.008. PMID: 30717904

[6] Zhao J Zhang Q Yang Y Yao J Liao L Dong J . High prevalence of thyroid carcinoma in patients with insulin resistance: a meta-analysis of case-control studies. Aging (Albany NY). (2020) 12:12848–61. doi: 10.18632/aging.203529, PMID: 34550096 PMC8507263

[7] Mohammed Hussein SM AbdElmageed RM . The relationship between type 2 diabetes mellitus and related thyroid diseases. Cureus. (2021) 13:e20697. doi: 10.7759/cureus.20697. PMID: 35106234 PMC8787293

[8] Xu N Liu H Wang Y Xue Y . Relationship between insulin resistance and thyroid cancer in Chinese euthyroid subjects without conditions affecting insulin resistance. BMC Endocr Disord. (2022) 22:58. doi: 10.1186/s12902-022-00943-6. PMID: 35255873 PMC8903656

[9] Kushchayeva Y Vasko V Jensen K Mendonca Torres MC Dwyer JE Auh S . Differentiated thyroid cancer in severe insulin resistance. AACE Endocrinol Diabetes. (2025) 12:229–40. doi: 10.1016/j.aed.2025.08.015. PMID: 41467137 PMC12744789

[10] Schlumberger M Leboulleux S . Current practice in patients with differentiated thyroid cancer. Nat Rev Endocrinol. (2021) 17:176–88. doi: 10.1038/s41574-020-00448-z. PMID: 33339988

[11] Fagin JA Wells SA . Biologic and clinical perspectives on thyroid cancer. N Engl J Med. (2016) 375:1054–67. doi: 10.1056/nejmra1501993. PMID: 27626519 PMC5512163

[12] Burrows N Babur M Resch J Williams KJ Brabant G . Hypoxia-inducible factor in thyroid carcinoma. J Thyroid Res. (2010) 2010:762905. doi: 10.4061/2011/762905. PMID: 21765994 PMC3134378

[13] He K Tao F Lu Y Fang M Huang H Zhou Y . The role of HK2 in tumorigenesis and development: potential for targeted therapy with natural products. Int J Med Sci. (2025) 22:790–805. doi: 10.7150/ijms.105553. PMID: 39991762 PMC11843137

[14] Riesco-Eizaguirre G Rodriguez I De la Vieja A Costamagna E Carrasco N Nistal M . The BRAF V600E oncogene induces transforming growth factor beta secretion leading to sodium iodide symporter repression. Cancer Res. (2009) 69:8317–25. doi: 10.1158/0008-5472.can-09-1248. PMID: 19861538

[15] Choe JH Mazambani S Kim TH Kim JW . Oxidative stress and the intersection of oncogenic signaling and metabolism in squamous cell carcinomas. Cells. (2021) 10:606. doi: 10.3390/cells10030606. PMID: 33803326 PMC8000417

[16] Ju SH Song M Lim JY Kang YE Yi HS Shong M . Metabolic reprogramming in thyroid cancer. Endocrinol Metab (Seoul). (2024) 39:425–44. doi: 10.3803/enm.2023.1802. PMID: 38853437 PMC11220218

[17] Li C Peng X Lv J Zou H Liu J Zhang K . SREBP1 as a potential biomarker predicts levothyroxine efficacy of differentiated thyroid cancer. BioMed Pharmacother. (2020) 123:109791. doi: 10.1016/j.biopha.2019.109791. PMID: 31887541

[18] Ferreira-da-Silva A Valacca C Rios E Pópulo H Soares P Sobrinho-Simões M . Mitochondrial dynamics protein Drp1 is overexpressed in oncocytic thyroid tumors and regulates cancer cell migration. PLoS One. (2015) 10:e0122308. doi: 10.1371/journal.pone.0122308. PMID: 25822260 PMC4379140

[19] Ren L Liu W Zheng J Wu Q Ai Z . The role of mitochondrial genome stability and metabolic plasticity in thyroid cancer. Biomedicines. (2025) 13:2599. doi: 10.3390/biomedicines13112599. PMID: 41301693 PMC12650402

[20] Grazioli S Pugin J . Mitochondrial damage-associated molecular patterns: from inflammatory signaling to human diseases. Front Immunol. (2018) 9:832. doi: 10.3389/fimmu.2018.00832. PMID: 29780380 PMC5946030

[21] Pasquali D Giacomelli L Pedicillo MC Conzo G Gentile G De Stefano IS . Tumor inflammatory microenvironment of the thyroid cancer: relationship between regulatory T-cell imbalance, and p-NFκB (p65) expression-a preliminary study. J Clin Med. (2023) 12:6817. doi: 10.3390/jcm12216817. PMID: 37959281 PMC10647421

[22] Wieser V Moschen AR Tilg H . Inflammation, cytokines and insulin resistance: a clinical perspective. Arch Immunol Ther Exp (Warsz). (2013) 61:119–25. doi: 10.1007/s00005-012-0210-1. PMID: 23307037

[23] Hotamisligil GS . Inflammation, meta-flammation and immunometabolic disorders. Nature. (2017) 542:177–85. doi: 10.1038/nature21363. PMID: 28179656

[24] Boucher J Kleinridders A Kahn CR . Insulin receptor signaling in normal and insulin-resistant states. Cold Spring Harb Perspect Biol. (2014) 6:a009191. doi: 10.1101/cshperspect.a009191. PMID: 24384568 PMC3941218

[25] Park JL Kim SK Jeon S Jung CK Kim YS . MicroRNA profile for diagnostic and prognostic biomarkers in thyroid cancer. Cancers (Basel). (2021) 13:632. doi: 10.3390/cancers13040632. PMID: 33562573 PMC7916038

[26] Ramírez-Moya J Santisteban P . An oncogenic role for microRNA-146b in the thyroid. Oncoscience. (2018) 5:155–6. doi: 10.18632/oncoscience.432. PMID: 30035174 PMC6049317

[27] Yang E Wang X Gong Z Yu M Wu H Zhang D . Exosome-mediated metabolic reprogramming: the emerging role in tumor microenvironment remodeling and its influence on cancer progression. Signal Transduct Target Ther. (2020) 5:242. doi: 10.1038/s41392-020-00359-5. PMID: 33077737 PMC7572387

[28] Dai J Su Y Zhong S Cong L Liu B Yang J . Exosomes: key players in cancer and metabolism. Cell Mol Life Sci. (2020) 77:4325–42. doi: 10.1038/s41392-020-00261-0. PMID: 32759948 PMC7406508

[29] Kim JW Kim JH Lee YJ . The role of adipokines in tumor progression and its association with obesity. Biomedicines. (2024) 12:97. doi: 10.3390/biomedicines12010097. PMID: 38255203 PMC10813163

[30] Zhao J Wen J Wang S Yao J Liao L Dong J . Association between adipokines and thyroid carcinoma: a meta-analysis of case-control studies. BMC Cancer. (2020) 20:788. doi: 10.1186/s12885-020-07299-x. PMID: 32819324 PMC7441682

[31] Belfiore A Pandini G Vella V Squatrito S Vigneri R . Insulin/IGF-I hybrid receptors play a major role in IGF-I signaling in thyroid cancer. Biochimie. (1999) 81:403–7. doi: 10.1016/s0300-9084(99)80088-1. PMID: 10401676

[32] Lin SL Lin CY Lee W Teng CF Shyu WC Jeng LB . Mini review: molecular interpretation of the IGF/IGF-1R axis in cancer treatment and stem cells-based therapy in regenerative medicine. Int J Mol Sci. (2022) 23:11781. doi: 10.3390/ijms231911781. PMID: 36233084 PMC9570316

[33] Ricarte-Filho JC Reichenberger ER Hinkle K Isaza A Bauer AJ Franco AT . TG-IGF1R: a novel receptor tyrosine kinase fusion oncogene in pediatric thyroid cancer. Thyroid. (2024) 34:1308–13. doi: 10.1089/thy.2024.0224, PMID: 39104254 PMC11958905

[34] Muniyappa R Montagnani M Koh KK Quon MJ . Cardiovascular actions of insulin. Endocr Rev. (2007) 28:463–91. doi: 10.1210/er.2007-0006. PMID: 17525361

[35] Ormazabal V Nair S Elfeky O Aguayo C Salomon C Zúñiga FA . Association between insulin resistance and the development of cardiovascular disease. Cardiovasc Diabetol. (2018) 17:122. doi: 10.1186/s12933-018-0762-4. PMID: 30170598 PMC6119242

[36] Huang TS Lee JJ Huang SY Cheng SP . Regulation of expression of sterol regulatory element-binding protein 1 in thyroid cancer cells. Anticancer Res. (2022) 42:2487–93. doi: 10.21873/anticanres.15727. PMID: 35489723

[37] Wan Y Li G Cui G Duan S Chang S . Reprogramming of thyroid cancer metabolism: from mechanism to therapeutic strategy. Mol Cancer. (2025) 24:74. doi: 10.1186/s12943-025-02263-4. PMID: 40069775 PMC11895238

[38] Ping P Ma Y Xu X Li J . Reprogramming of fatty acid metabolism in thyroid cancer: potential targets and mechanisms. Chin J Cancer Res. (2025) 37:227–49. doi: 10.21147/j.issn.1000-9604.2025.02.09. PMID: 40353071 PMC12062987

[39] Bornfeldt KE Tabas I . Insulin resistance, hyperglycemia, and atherosclerosis. Cell Metab. (2011) 14:575–85. doi: 10.1016/j.cmet.2011.07.015. PMID: 22055501 PMC3217209

[40] Lopaschuk GD Ussher JR Folmes CDL Jaswal JS Stanley WC . Myocardial fatty acid metabolism in health and disease. Physiol Rev. (2010) 90:207–58. doi: 10.1152/physrev.00015.2009. PMID: 20086077

[41] Strathearn LS Stepanov AI Font-Burgada J . Inflammation in primary and metastatic liver tumorigenesis-under the influence of alcohol and high-fat diets. Nutrients. (2020) 12:933. doi: 10.3390/nu12040933. PMID: 32230953 PMC7230665

[42] Jabbar A Pingitore A Pearce SHS Zaman A Iervasi G Razvi S . Thyroid hormones and cardiovascular disease. Nat Rev Cardiol. (2017) 14:39–55. doi: 10.1038/nrcardio.2016.174. PMID: 27811932

[43] Ringel MD Sosa JA Baloch Z Bischoff L Bloom G Brent GA . 2025 American Thyroid Association management guidelines for adult patients with differentiated thyroid cancer. Thyroid. (2025) 35:841–985. doi: 10.1177/10507256251363120. PMID: 40844370 PMC13090833

[44] Yang X Guo N Gao X Liang J Fan X Zhao Y . Meta-analysis of TSH suppression therapy and the risk of cardiovascular events after thyroid cancer surgery. Front Endocrinol (Lausanne). (2022) 13:991876. doi: 10.3389/fendo.2022.991876. PMID: 36619576 PMC9814721

[45] Uludag M Unlu MT Cetinoglu I Caliskan O Aygun N . What has changed in the 2025 American Thyroid Association management guidelines for adult patients with differentiated thyroid cancer? Part 2: postoperative initial treatment. Sisli Etfal Hastan Tip Bul. (2025) 59:273–83. doi: 10.14744/semb.2025.30906. PMID: 41573618 PMC12821094

[46] Kushchayeva Y Kushchayev S Jensen K Brown RJ . Impaired glucose metabolism, anti-diabetes medications, and risk of thyroid cancer. Cancers (Basel). (2022) 14:555. doi: 10.3390/cancers14030555. PMID: 35158824 PMC8833385

[47] Nagueh SF Smiseth OA Appleton CP Byrd BF 3rd Dokainish H Edvardsen T . Recommendations for the evaluation of left ventricular diastolic function by echocardiography: an update from the American Society of Echocardiography and the European Association of Cardiovascular Imaging. J Am Soc Echocardiogr. (2016) 29:277–314. doi: 10.1016/j.echo.2016.01.011, PMID: 27037982

[48] Zhang H Yang Y Gao C Tian L . Effect of thyroid-stimulating hormone suppression therapy on cardiac structure and function in patients with differentiated thyroid cancer after thyroidectomy: a systematic review and meta-analysis. Endocr Pract. (2024) 30:177–86. doi: 10.1016/j.eprac.2023.11.006. PMID: 38007181

[49] Klein Hesselink EN Klein Hesselink MS de Bock GH Gansevoort RT Bakker SJL Vredeveld EJ . Long-term cardiovascular mortality in patients with differentiated thyroid carcinoma: an observational study. J Clin Oncol. (2013) 31:4046–53. doi: 10.1200/jco.2013.49.1043. PMID: 24101052

[50] Praw SS Gigliotti BJ Tessnow A Kang H Margulies DJ . Executive summary of the 2025 American Thyroid Association management guidelines for adult patients with differentiated thyroid cancer. Thyroid. (2025) 35:1214–20. doi: 10.1177/10507256251390877. PMID: 41173539

[51] Wang X Ye Y Amdulla M Ren C Liu Y Ni S . The necessity of thyroid-stimulating hormone suppression therapy for low-risk differentiated thyroid carcinoma following hemithyroidectomy: A systematic review and meta-analysis. Heliyon. (2024) 10:e40574. doi: 10.1016/j.heliyon.2024.e40574. PMID: 39654735 PMC11626026

[52] Leboulleux S Bournaud C Chougnet CN Lamartina L Zerdoud S Do Cao C . Thyroidectomy without radioiodine in patients with low-risk thyroid cancer. N Engl J Med. (2022) 386:923–32. doi: 10.1056/nejmoa2111953. PMID: 35263518

[53] Klubo-Gwiezdzinska J Jensen K Costello J Patel A Hoperia V Bauer A . Metformin inhibits growth and decreases resistance to anoikis in medullary thyroid cancer cells. Endocr Relat Cancer. (2012) 19:447–56. doi: 10.1530/erc-12-0046. PMID: 22389381

[54] Chen G Xu S Renko K Derwahl M . Metformin inhibits growth of thyroid carcinoma cells, suppresses self-renewal of derived cancer stem cells, and potentiates the effect of chemotherapeutic agents. J Clin Endocrinol Metab. (2012) 97:E510–20. doi: 10.1210/jc.2011-1754. PMID: 22278418

[55] García-Sáenz M Lobaton-Ginsberg M Ferreira-Hermosillo A . Metformin in differentiated thyroid cancer: Molecular pathways and its clinical implications. Biomolecules. (2022) 12:574. doi: 10.3390/biom12040574, PMID: 35454163 PMC9029304

[56] Drucker DJ . The cardiovascular biology of glucagon-like peptide-1. Cell Metab. (2016) 24:15–30. doi: 10.1016/j.cmet.2016.06.009. PMID: 27345422

[57] Marso SP Daniels GH Brown-Frandsen K Kristensen P Mann JFE Nauck MA . Liraglutide and cardiovascular outcomes in type 2 diabetes (LEADER trial). N Engl J Med. (2016) 375:311–22. doi: 10.1056/nejmoa1603827. PMID: 27295427 PMC4985288

[58] Verma S McMurray JJ . SGLT2 inhibitors and mechanisms of cardiovascular benefit: a state-of-the-art review. Diabetologia. (2018) 61:2108–17. doi: 10.1007/s00125-018-4670-7. PMID: 30132036

[59] McMurray JJV Solomon SD Inzucchi SE Køber L Kosiborod MN Martinez FA . Dapagliflozin in patients with heart failure and reduced ejection fraction. N Engl J Med. (2019) 381:1995–2008. doi: 10.1056/nejmoa1911303. PMID: 31535829

[60] Seidu S Alabraba V Davies S Newland-Jones P Fernando K Bain SC . SGLT2 inhibitors - The new standard of care for cardiovascular, renal and metabolic protection in type 2 diabetes: A narrative review. Diabetes Ther. (2024) 15:1099–124. doi: 10.1007/s13300-024-01550-5. PMID: 38578397 PMC11043288

[61] Cesaro A De Michele G Fimiani F Acerbo V Scherillo G Signore G . Visceral adipose tissue and residual cardiovascular risk: a pathological link and new therapeutic options. Front Cardiovasc Med. (2023) 10:1187735. doi: 10.3389/fcvm.2023.1187735. PMID: 37576108 PMC10421666

[62] Shin J Heo SJ Lee YJ Kang SW Kwon YJ Lee JW . Mediterranean diet adherence and one-year metabolic changes in patients with papillary thyroid cancer: An observational study. Nutrients. (2025) 17:3420. doi: 10.3390/nu17213420. PMID: 41228493 PMC12611023

[63] Aziz T Niraj MK Kumar S Kumar R Parveen H . Effectiveness of omega-3 polyunsaturated fatty acids in non-alcoholic fatty liver disease: A systematic review and meta-analysis. Cureus. (2024) 16:e68002. doi: 10.7759/cureus.68002. PMID: 39347373 PMC11428178

[64] Pham NHT Joglekar MV Wong WKM Nassif NT Simpson AM Hardikar AA . Short-chain fatty acids and insulin sensitivity: a systematic review and meta-analysis. Nutr Rev. (2024) 82:193–209. doi: 10.1093/nutrit/nuad042. PMID: 37290429 PMC10777678

[65] Portincasa P Bonfrate L Vacca M De Angelis M Farella I Lanza E . Gut microbiota and short chain fatty acids: Implications in glucose homeostasis. Int J Mol Sci. (2022) 23:1105. doi: 10.3390/ijms23031105. PMID: 35163038 PMC8835596

[66] López-Gil JF García-Hermoso A Martínez-González MÁ Rodríguez-Artalejo F . Mediterranean diet and cardiometabolic biomarkers in children and adolescents: A systematic review and meta-analysis. JAMA Netw Open. (2024) 7:e2421976. doi: 10.1001/jamanetworkopen.2024.21976. PMID: 38995643 PMC11245727

[67] de Cabo R Mattson MP . Effects of intermittent fasting on health, aging, and disease. N Engl J Med. (2019) 381:2541–51. doi: 10.1056/nejmra1905136. PMID: 31881139

[68] Richter EA Hargreaves M . Exercise, GLUT4, and skeletal muscle glucose uptake. Physiol Rev. (2013) 93:993–1017. doi: 10.1152/physrev.00038.2012. PMID: 23899560

[69] Campbell KL Winters-Stone KM Wiskemann J May AM Schwartz AL Courneya KS . Exercise guidelines for cancer survivors: Consensus statement from international multidisciplinary roundtable. Med Sci Sports Exerc. (2019) 51:2375–90. doi: 10.1249/mss.0000000000002116. PMID: 31626055 PMC8576825

[70] Ferrante M Distefano G Distefano C Copat C Grasso A Oliveri Conti G . Benefits of physical activity during and after thyroid cancer treatment on fatigue and quality of life: A systematic review. Cancers (Basel). (2022) 14:3657. doi: 10.3390/cancers14153657. PMID: 35954324 PMC9367318

[71] Hivert MF Sullivan LM Shrader P Fox CS Nathan DM D’Agostino RB Sr . The association of tumor necrosis factor alpha receptor 2 and tumor necrosis factor alpha with insulin resistance and the influence of adipose tissue biomarkers in humans. Metabolism. (2010) 59:540–6. doi: 10.1016/j.metabol.2009.08.017. PMID: 19846171 PMC2843788

[72] Yu J Kaur R Ayeni FE Eslick GD Edirimanne S . Cardiovascular outcomes of differentiated thyroid cancer patients on long term TSH suppression: A systematic review and meta-analysis. Horm Metab Res. (2023) 55:379–87. doi: 10.1055/a-2084-3408. PMID: 37295414

